# Clinical impact of respiratory syncytial virus infection on children hospitalized for pertussis

**DOI:** 10.1186/s12879-021-05863-9

**Published:** 2021-02-09

**Authors:** Ruimu Zhang, Jikui Deng

**Affiliations:** Department of Infectious Diseases, Shenzhen Children’s Hospital, Shenzhen, China

**Keywords:** Pertussis, Respiratory syncytial virus, Children

## Abstract

**Background:**

Although Respiratory syncytial virus (RSV) is one of the common pathogens in children with pertussis and viral coinfection, the clinical impact of RSV infection on pertussis remains unclear. We compared clinical characteristics and sought differences between infants with single *Bordetella pertussis* (*B. pertussis*) infection and those with RSV coinfection.

**Methods:**

We enrolled 80 patients with pertussis who were hospitalized in Shenzhen Children’s Hospital from January 2017 to December 2019. Respiratory tract samples were tested for *B. pertussis* with real-time polymerase chain reaction and respiratory viruses with immunofluorescence assay. Clinical data were obtained from hospital records and collected using a structured questionnaire.

**Results:**

Thirty-seven of 80 patients had *B. pertussis* infection alone (pertussis group) and 43 had RSV-pertussis coinfection (coinfection group). No significant differences were found with regard to sex, body weight, preterm birth history, pertussis vaccination, symptoms, presence of pneumonia, or lymphocyte count between the 2 groups. Univariate analysis showed patients with RSV coinfection were older (median, 4.57 months vs 4.03 months, *p* = 0.048); more commonly treated with β-lactam antibiotics (21% vs 5%, *p* = 0.044); had higher rates of wheezes (40% vs 14%, *p* = 0.009) and rales (35% vs 14%, *p* = 0.028) on chest auscultation, a higher rate of readmission (40% vs 11%, *p* = 0.004), and a longer hospital stay (median, 10 days vs 7 days, *p* = 0.002). In the further binary logistic regression analysis, patients with RSV coinfection had higher rates of wheezes (OR = 3.802; 95% CI: 1.106 to 13.072; *p* = 0.034) and readmission (OR = 5.835; 95% CI: 1.280 to 26.610; *p* = 0.023).

**Conclusions:**

RSV coinfection increases readmission rate in children hospitalized for pertussis. RSV infection should be suspected when wheezes are present on auscultation of the chest in these patients. Early detection of RSV may avoid unnecessary antibiotic use.

## Background

Early in 1973, Respiratory syncytial virus (RSV) and *Bordetella pertussis* (*B. pertussis*) coinfection has been noticed in patients with pertussis [1]. A report in 1996 described two young infants with respiratory failure caused by dual infection of RSV and *B. pertussis*, which highlighted the importance of viral detection in pertussis [2].

Studies in recent years have confirmed that RSV and *B. pertussis* coinfection is common in children [3–7]. In previous studies, comparisons were mainly made between viral bronchiolitis and viral-pertussis coinfection, rather than pertussis and viral-pertussis coinfection, and no differences were found in clinical symptoms [3, 5]. Comparison has not been made between pertussis and specific RSV-pertussis coinfection, and the clinical impact of RSV infection on pertussis remains unclear. Therefore, we designed this study to report the clinical characteristics of RSV-pertussis coinfection and explored the clinical impact of RSV infection on children hospitalized for pertussis.

## Methods

### Study design

This study was a retrospective, observational study conducted at Shenzhen Children’s Hospital, a 1300-bed tertiary care facility in Shenzhen, China. The study population consisted of children hospitalized from January 2017 to December 2019 with positive *B. pertussis* polymerase chain reaction (PCR) and/or culture tests. Patients with a negative RSV PCR test and negative immunofluorescence assay of 7 respiratory viruses were included in the pertussis group. Patients who met both of the following criteria were included in the RSV coinfection group: a positive RSV test using PCR or immunofluorescence assay; and a negative immunofluorescence assay of the other 6 respiratory viruses except for RSV. Patients who met one of the following criteria were excluded: confirmed or suspected bacterial infection other than pertussis (positive culture of bacteria other than B. pertussis, elevated C-reactive protein, elevated procalcitonin, or presence of neutrophilia); newborns; infection of HIV; leukemia; known or suspected active tuberculosis; receiving immunosuppressive agents; immunodeficiency; and chronic conditions (malnutrition, congenital heart disease, or chronic lung disease).

### Data collection and management

Demographic information (age, sex, preterm birth history, and vaccination status), symptoms and signs (cough, inspiratory whooping, cyanosis, and auscultation of the chest), laboratory results (hematology and pathogen tests), chest imaging results (chest x-ray and/or computed tomography), antibiotic treatment, hospitalization information (readmission due to relapse and length of hospital stay), and outcome (survival, death, recovery or discharged against medical advice) were recorded.

This study was approved by the ethics committee of Shenzhen Children’s Hospital, and written informed consent was obtained from patients’ guardians.

### Chest images

Chest radiographs or computed tomography images were read by 2 radiologists. The diagnosis of pneumonia was made if infiltrate, consolidation, or atelectasis was present in chest images.

### Sample management

Two nasopharyngeal samples and two oropharyngeal samples were obtained per patient on admission. All of the respiratory tract samples were transported to the laboratory within 4 h.

### *Bordetella pertussis* detection by culture and PCR

Nasopharyngeal swab samples were used for *B. pertussis* culture and PCR. Samples were cultured on charcoal agar plates (OXOID, United Kingdom) supplemented with 10% defibrinated sheep blood and cephalexin. The plates were incubated in a humidified incubator at 37 °C for up to 7 days and inspected daily. Real-time PCR was performed using “*Bordetella pertussis* real-time fluorescence PCR detection kit” (DaAn Gene Co. Ltd., Guangzhou, China), following the manufacturer’s instructions.

### Respiratory virus detection

Oropharyngeal swab samples were tested for respiratory viruses. Immunofluorescence assay of 7 respiratory viruses (influenza A, influenza B, RSV, adenovirus, parainfluenza 1, parainfluenza 2, and parainfluenza 3 viruses) was performed using “D3® UltraTM DFA Respiratory Virus Screening and ID Kit” (Diagnostic Hybrids, Inc. USA). RSV Real-time PCR was performed using “the respiratory syncytial virus real-time fluorescence PCR detection kit” (DaAn Gene Co. Ltd., Guangzhou, China), following the manufacturer’s instructions.

### Statistical analysis

We did a univariate correlation analysis of clinical variables to determine significant differences in clinical characteristics between the 2 groups. Chi-square test was used for categorical variables. The Student *t* test or Mann-Whitney test was used for continuous variables, as appropriate. A binary logistic regression analysis was also performed to control confounding effects. Continuous variables were summarized as mean (standard deviation) if they were normally distributed and as median (interquartile range, IQR) if they had a skewed distribution. Data analysis was performed by SPSS 26.0 software. All *P*-values were two-tailed, and *P* < 0.05 was considered to indicate statistical significance.

## Results

**Fig. 1:**
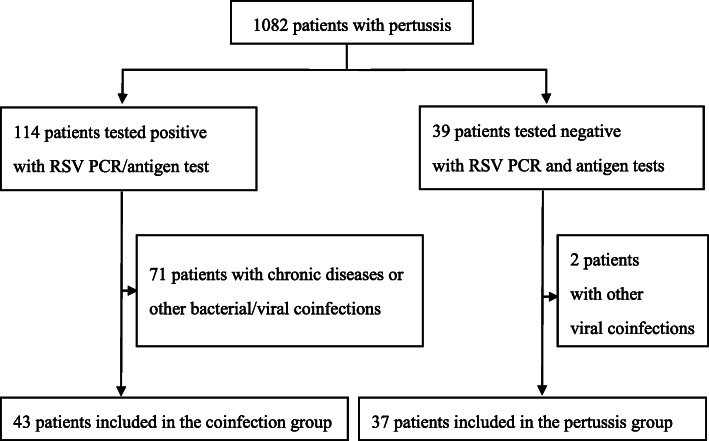
Study profile

Between January 2017 and December 2019, 1082 children were hospitalized for pertussis (Fig. 1). Immunofluorescence assay of respiratory viruses was performed in 1036 patients and RSV PCR was performed in 46 patients. In total, 114 (10.54%) children tested positive for RSV. Of the 80 children (median age 4.30 months, range 1.17–34.87 months, 42 boys) enrolled in this study, 37 were included in the pertussis group and 43 were included in the RSV coinfection group (Table 1). RSV cases were mostly detected in August (15 children) and September (12 children), while fewer than 5 RSV-pertussis coinfection patients were observed in each of the other months. In the pertussis group, 33 children had chest image examinations, of which 16 indicated pneumonia. In the coinfection group, 42 children had chest image examinations, of which 16 indicated pneumonia. While all of the patients received macrolide treatment, 2 patients in the pertussis group and 9 in the coinfection group received additional β-lactam antibiotic treatment. According to medical records, reasons for β-lactam antibiotic use were as follows: presence of rales on chest auscultation in 9 patients; pneumonia in 1 patient; and excessive sputum in 1 patient. In the coinfection group, β-lactam antibiotics were discontinued when positive RSV test results were available. One child from the coinfection group was admitted to intensive care unit (ICU). None of the patients were endotracheal intubated. All of the patients were discharged with alleviation of symptoms but 21 patients were readmitted due to a relapse of symptoms. The interval between discharge and readmission ranged from 1 to 24 days.

**Table 1:** Clinical characteristics of children with single *B. pertussis* infection and children with *B. pertussis*-RSV coinfection

Characteristics	Pertussis Group (*n* = 37)	Coinfection Group (*n* = 43)	*P* value
Demographics			
Male, n (%)	18 (49%)	24 (59%)	0.522
Age, months, median (IQR)	4.03 (1.85–5.05)	4.57 (3.40–6.03)	0.048
Body weight, kg, median (IQR)	6.60 (5.40–7.75)	6.70 (5.80–7.80)	0.732
Preterm birth, n (%)	3 (8%)	7 (16%)	0.271
*Pertussis* vaccination, n (%)	18 (49%)	24 (59%)	0.522
Symptoms and signs			
Paroxysms of coughing, n (%)	37 (100%)	43 (100%)	–
Inspiratory whooping, n (%)	11 (30%)	19 (44%)	0.183
Cyanosis, n (%)	17 (46%)	19 (44%)	0.875
Wheezes on auscultation, n (%)	5 (14%)	17 (40%)	0.009
Rales on auscultation, n (%)	5 (14%)	15 (35%)	0.028
Pneumonia, n (%)	16 (43%)	16 (37%)	0.583
Laboratory tests			
WBC count, 10^9^/L, median (IQR)	16.19 (13.37–19.69)	14 .04 (11.92–18.43)	0.110
Lymphocyte count, 10^9^/L, median (IQR)	10.38 (8.50–14.68)	9.29 (8.27–13.76)	0.364
Treatment and hospitalization			
β-lactam antibiotic use, n (%)	2 (5%)	9 (21%)	0.044
ICU admission, n	1	0	–
Readmission, n (%)	4 (11%)	17 (40%)	0.004
Hospital stay, days, median (IQR)	7 (6–10)	10 (7–15)	0.002

No significant differences were found with regard to sex, body weight, preterm birth history, pertussis vaccination, symptoms, presence of pneumonia, or lymphocyte count between the 2 groups. In univariate analysis, patients with RSV coinfection were older (median, 4.57 months vs 4.03 months, *p* = 0.048); received more β-lactam antibiotic treatment (21% vs 5%, *p* = 0.044); had higher rates of wheezes (40% vs 14%, *p* = 0.009) and rales (35% vs 14%, *p* = 0.028) on chest auscultation, a higher rate of readmission (40% vs 11%, *p* = 0.004), and a longer hospital stay (median, 10 days vs 7 days, *p* = 0.002).

We did a binary logistic regression analysis including the following variables: age, β-lactam antibiotic treatment, wheezes, rales, readmission, and length of hospital stay (Table 2). In binary logistic regression analysis, patients with RSV coinfection had higher rates of wheezes (OR = 3.802; 95% CI: 1.106 to 13.072; *p* = 0.034) and readmission (OR = 5.835; 95% CI: 1.280 to 26.610; *p* = 0.023).

**Table 2:** Comparison between children with single *B. pertussis* infection and children with *B. pertussis*-RSV coinfection in binary logistic regression analysis

Characteristics	Pertussis Group (*n* = 37)	Coinfection Group (*n* = 43)	*P* value	Odds Ratio (95% CI)
Age, months, median (IQR)	4.03 (1.85–5.05)	4.57 (3.40–6.03)	0.753	1.001 (0.997–1.004)
Wheezes on auscultation, n (%)	5 (14%)	17 (40%)	0.034	3.802 (1.106–13.072)
Rales on auscultation, n (%)	5 (14%)	15 (35%)	0.192	2.450 (0.637–9.424)
β-lactam antibiotic use, n (%)	2 (5%)	9 (21%)	0.133	3.925 (0.660–23.337)
Readmission, n (%)	4 (11%)	17 (40%)	0.023	5.835 (1.280–26.610)
Hospital stay, days, median (IQR)	7 (6–10)	10 (7–15)	0.756	1.020 (0.899–1.159)

## Discussion

RSV has been recognized as a common pathogen in children with pertussis and viral coinfection for years [3–7]. In previous studies, RSV coinfection rates among patients with pertussis varied from 3.80 to 6.47% [6, 8]. In our study, 114 patients tested positive for RSV among the 1082 children hospitalized for pertussis and the coinfection rare was 10.54%. The peak RSV activity ranged from September to October, basically consistent with the RSV seasonality in other countries of the northern hemisphere [9].

For early recognition of RSV coinfection, we included the variables of wheezes and rales on chest auscultation in the analysis. In our clinical practice, although wheezes and rales can sometimes be heard in patients with pertussis, they are more common in patients with RSV infection. In our study, wheezes were heard in 14% of children in the pertussis group and 40% in the coinfection group, while rales were heard in 14% of children in the pertussis group and 35% in the coinfection group. The value of wheezes was also confirmed in logistic regression analysis. This indicates chest auscultation, especially wheezes, can be helpful in the early recognition of RSV coinfection before laboratory test results are available.

As β-lactam antibiotics may be prescribed to children with rales on chest auscultation, we also investigated the antibiotic use in this study. In a report of 188 children with RSV lower respiratory tract infections, one-third of children were treated unnecessarily with antibiotics [10]. Another study suggested that rapid testing for respiratory viruses was associated with less antibiotic use among patients with influenza-like illness [11]. In our study, while all of the patients received macrolide treatment, 2 patients in the pertussis group and 9 in the coinfection group received additional β-lactam antibiotic treatment, principally because of presence of rales on chest auscultation. In the coinfection group, β-lactam antibiotics were discontinued when positive RSV test results were available and patients still had alleviation of symptoms, indicating unnecessary β-lactam antibiotic use. Early detection of RSV may avoid unnecessary antibiotic use.

In a previous study, length of hospital stay was not significantly different between patients with pertussis and patients with viral-pertussis coinfection. Viruses detected in patients with coinfection included rhinovirus, coronavirus, and respiratory syncytial virus. Comparison was not made by single respiratory virus, and readmission rate was not included in the analysis [8]. In our study, readmission rate and length of hospital stay were compared between patients with pertussis and those with specific RSV coinfection. Upon statistical analysis, patients with RSV coinfection had a higher readmission rate, revealing the clear impact of RSV coinfection. Immunoprophylactic agents for RSV, such as palivizumab and nirsevimab, are currently only recommended for children at high risk of RSV disease [12, 13]. The prophylactic value of these agents for RSV in children with pertussis is unknown.

This study has limitations due to its retrospective nature and small sample size. In order to reveal the single impact of RSV infection on pertussis, we excluded patients with underlying chronic diseases and patients with another viral/bacterial coinfection, which resulted in the small sample size of the RSV coinfection group. In order to make sure patients in the pertussis group were not coinfected by RSV, we only enrolled patients who had tested negative by RSV PCR and antigen. As few patients were tested by RSV PCR, the sample size of the pertussis group was also small. A prospective study which screens every pertussis patient for coinfection pathogens by a multiplex PCR assay may detect pathogens better and increase the sample size.

## Conclusions

RSV coinfection increases readmission rate in children hospitalized for pertussis. RSV infection should be suspected when wheezes are present on auscultation of the chest in these patients. Early detection of RSV may avoid unnecessary antibiotic use.

## Data Availability

The datasets used in the current study are available from the corresponding author on reasonable request.

## Abbreviations

*B. pertussis*: *Bordetella pertussis*
IQR: Interquartile range
PCR: Polymerase chain reaction
RSV: Respiratory syncytial virus
